# A Case of Spinal Cord Infarction With Pansensory Deficit: Discussing the Possible Etiology

**DOI:** 10.7759/cureus.71880

**Published:** 2024-10-19

**Authors:** Rina Izumi, Koji Hayashi, Yuka Nakaya, Asuka Suzuki, Naoko Takaku, Mamiko Sato, Yasutaka Kobayashi

**Affiliations:** 1 Department of Rehabilitation Medicine, Fukui General Hospital, Fukui, JPN; 2 Graduate School of Health Science, Fukui Health Science University, Fukui, JPN

**Keywords:** anterior spinal cord infarction, owl-eye appearance, owl's eye sign, position sense, sensory impairment, : spinal cord infarction, vibration sense

## Abstract

Spinal cord infarction (SCI) is a rare vascular condition that can lead to the sudden onset of myelopathy, manifesting as paraplegia, bladder and bowel dysfunction, and/or sensory impairments. The primary arteries supplying the spinal cord are the anterior spinal artery (ASA) and the posterior spinal artery (PSA). The ASA mainly provides blood to the anterior two-thirds of the spinal cord, excluding the posterior columns, while the PSA primarily supplies the posterior one-third, which includes the posterior columns. It is relatively uncommon for vascular SCI to result in complete sensory loss, as the area supplied by the ASA is mainly associated with superficial sensation, while the PSA is related to deep sensation. In this report, we describe a case of SCI with pansensory deficits and explore the potential causes of pansensory loss in SCI.

The patient was a 51-year-old healthy woman who experienced sudden lower back discomfort, progressing to bilateral lower limb weakness within 30 minutes, accompanied by urinary retention after lifting a heavy object. She was transferred to the hospital with stable vitals except for high blood pressure. A neurological examination revealed significant weakness in her lower limbs, hypesthesia below the Th10 level, bathyanesthesia, and areflexia. Spinal MRI showed hyperintensities at the Th11-Th12 levels, leading to a diagnosis of spinal cord infarction. She was treated with methylprednisolone, heparin, and rehabilitation. Over time, her muscle strength and sensory loss improved, though dysesthesia persisted. After 80 days of treatment and rehabilitation, she was able to walk independently with assistance and was discharged.

## Introduction

Spinal cord infarction (SCI) is a rare vascular event, accounting for only 1% of all strokes [1,2]. SCI can be classified into several types based on the affected part of the spinal cord or the responsible arteries: anterior spinal artery (ASA) infarction, anterior unilateral infarction or pseudopoliomyelitic form, posterior spinal artery (PSA) infarction, posterior unilateral infarction, central infarction, and transverse infarction [3]. Depending on the affected area, various symptoms may appear, including paraplegia, bladder and rectal dysfunction, superficial sensory disorders, and deep sensory disorders [3,4]. Among these symptoms, pansensory deficit is relatively rare. In this report, we describe a case of SCI with a pansensory deficit and, based on previous reports, discuss the potential etiologies related to the development of pansensory deficits in SCI.

## Case presentation

A 51-year-old previously healthy woman presented with a sudden onset of lower back discomfort, which progressed to bilateral lower limb weakness within 30 minutes, resulting in gait difficulties. Immediately before onset, she lifted a heavy object from a crouched position. She also developed urinary retention. She was transferred to our hospital from another facility. Upon arrival, her vital signs were stable except for elevated blood pressure (185/110 mmHg). Physical examination revealed a slightly cold sensation in the left toe, but both dorsalis pedis arteries were intact and palpable. The neurological examination revealed clear consciousness with no cranial nerve abnormalities. Muscle strength in the upper limbs was normal, but there was significant weakness in the lower extremities, with muscle strength reduced to grades 1-2: iliopsoas (1/2), quadriceps (1/2), hamstrings (1/2), and both anterior and posterior leg muscles (1/2) on the right and left sides. There was severe hypesthesia, predominantly on the left side, affecting both superficial sensation below the Th10 level (below the umbilicus) and deep sensation, including vibration and proprioception in the toes, more pronounced on the right. The lower extremities showed areflexia, and pathological reflexes were absent. Blood tests revealed elevated red blood cells, white blood cells, hemoglobin, triglycerides, and D-dimer levels (Table 1). Cerebrospinal fluid (CSF) analysis was unremarkable.

**Table 1:** The results of the blood test on admission.

Inspection items	Result	Reference range
White blood cell count	101×10^2^ /μl	(3300–8600)
Red blood cell count	570×10⁴ /μl	(386–492×10⁴)
Hemoglobin	15.8 g/dl	(11.6–33.4)
Blood platelet	30.0×10⁴ /μl	(15.8–34.8)
Total protein	7.8 g/dl	(6.6–8.1)
Albumin	4.8 g/dl	(4.1–5.1)
Blood urea nitrogen	6.0 mg/dl	(8.0–20.0)
Creatinine	0.44 mg/dl	(0.46–0.79)
Total bilirubin	0.8 mg/dl	(0.4-1.2)
Aspartate aminotransferase	20 U/l	(13–30)
Alanine aminotransferase	13 U/l	(7–30)
Alkaline phosphatase	60 U/l	(38–113)
Lactate dehydrogenase	186 U/l	(124–222)
γ-glutamyltransferase	40 U/l	(13-64)
Creatine phosphokinase	69 U/l	(41–153)
Sodium	139 mmol/l	(138–145)
Potassium	3.8 mmol/l	(3.6–4.8)
Chlorine	100 mmol/l	(101–108)
C-reactive protein	0.14 mg/dl	(0.00–0.14)
Prothrombin time-international normalized ratio	0.92	(0.85–1.15)
Activated partial thromboplastin time	27.9 sec	(25.0–40.0)
D-dimmer	1.4 μg/ml	(<1.0)
Triglyceride	309 mg/dl	(30–149)
High-density lipoprotein cholesterol	55 mg/dl	(40–119)
Low-density lipoprotein cholesterol	127 mg/dl	(70–139)
Antinuclear antibody	<1:40 titer	(<1:40)
Anticardiolipin antibody	<4 U/l	(<4)
anti-β2-glycoprotein I antibody	<1.3 U/ml	(<1.3)
Proteinase 3-specific antineutrophil cytoplasmic antibody	<0.6 IU/ml	(<0.6)
Myeloperoxidase-specific antineutrophil cytoplasmic antibody	<0.2 IU/ml	(<0.2)

**Figure 1:**
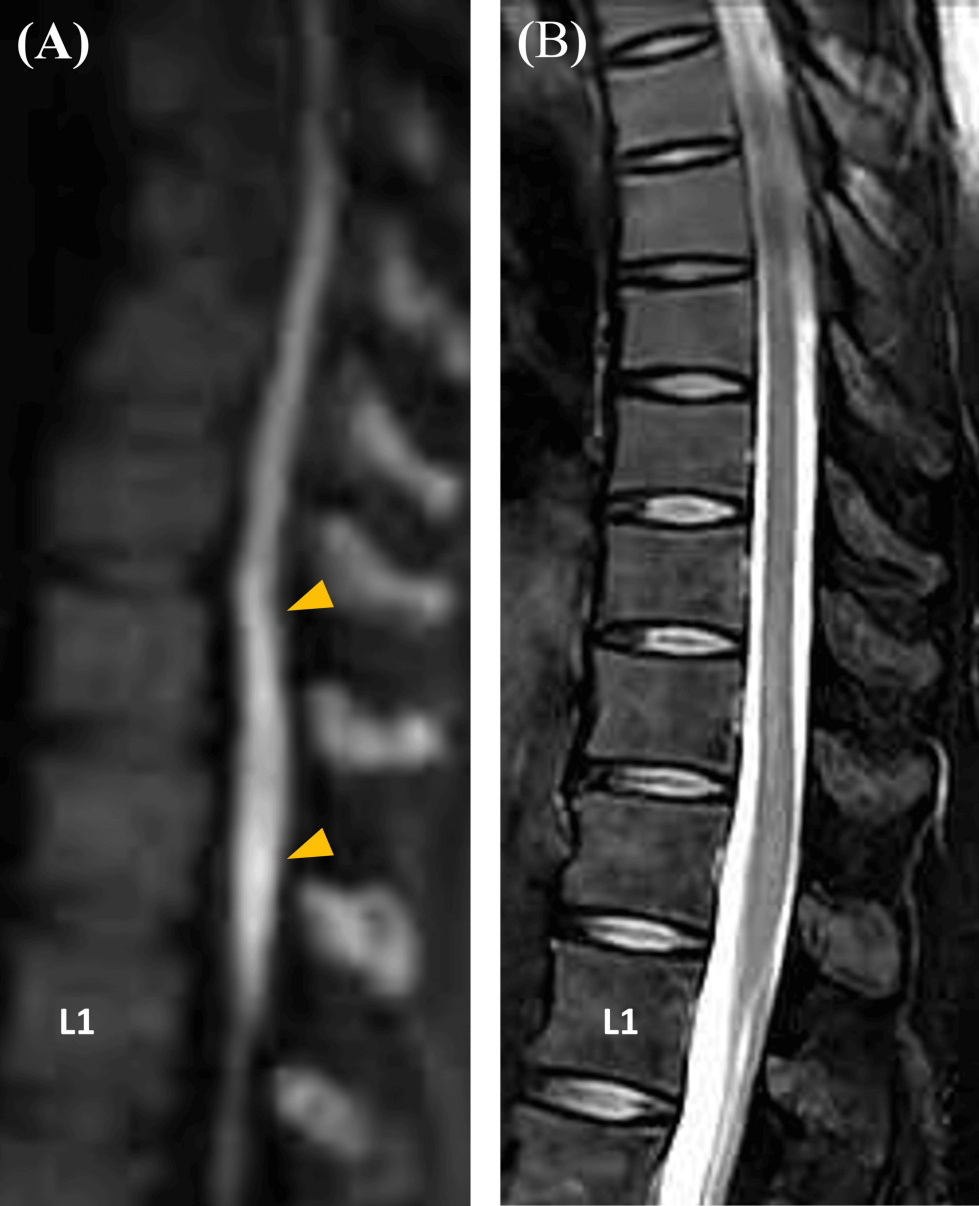
The results of thoracic magnetic resonance imaging (MRI) on diffusion-weighted and T2-weighted imaging on admission. A: diffusion-weighted thoracic MRI showing hyperintensities in the spinal cord at the Th11 and Th12 levels; B: T2-weighted MRI showing no hyperintensity in the spinal cord

**Figure 2:**
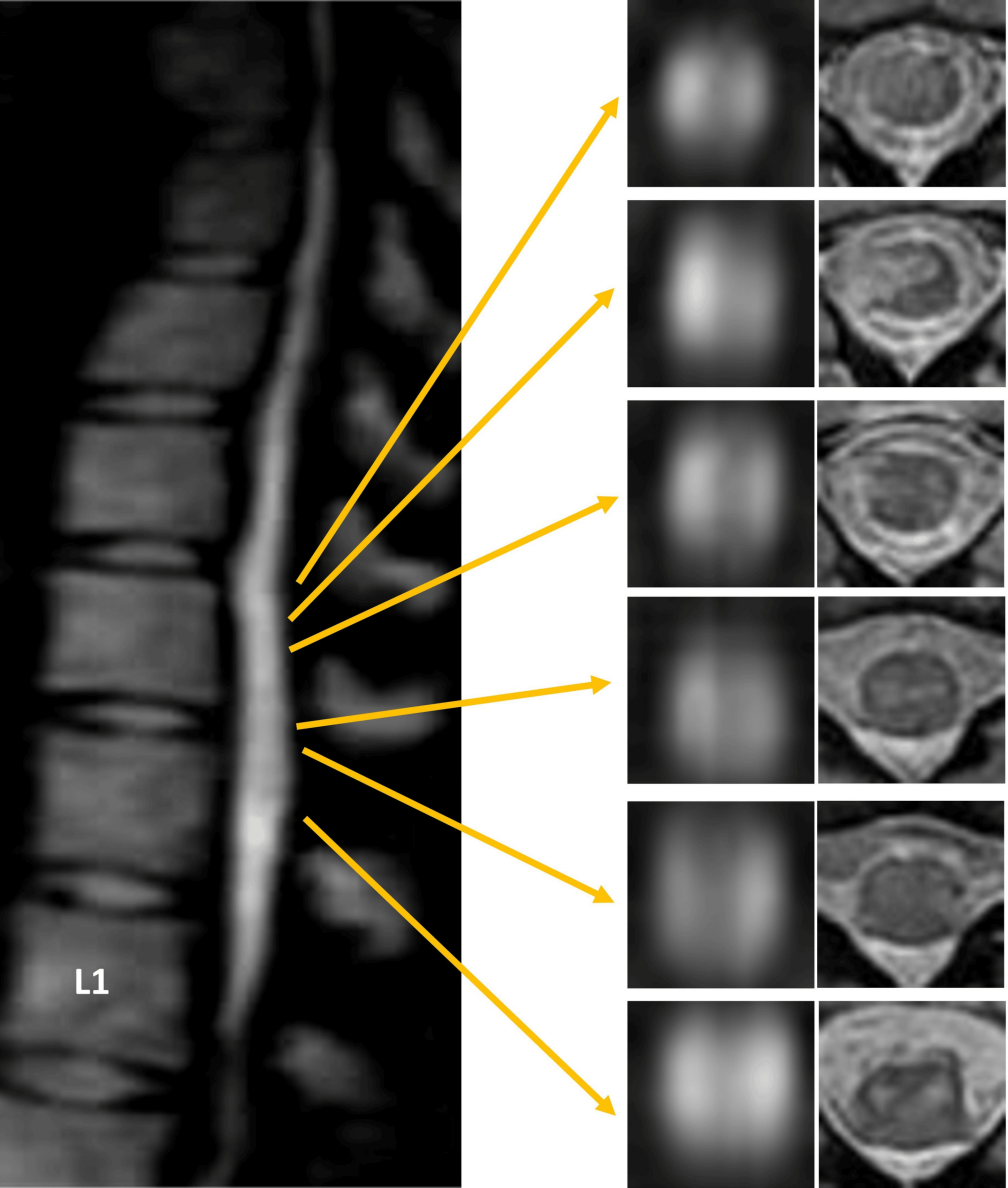
The result of thoracic MRI on diffusion-weighted and T2-weighted imaging on day three. Diffusion-weighted and T2-weighted thoracic MRI show hyperintensity in the spinal cord at the Th10 to Th12 levels. Axial sections of diffusion-weighted imaging reveal the "owl sign" between the Th10 and Th12 levels. The sagittal section is shown using diffusion-weighted imaging. The axial section to the left also shows diffusion-weighted imaging. The axial section on the right demonstrates T2-weighted imaging.

**Figure 3:**
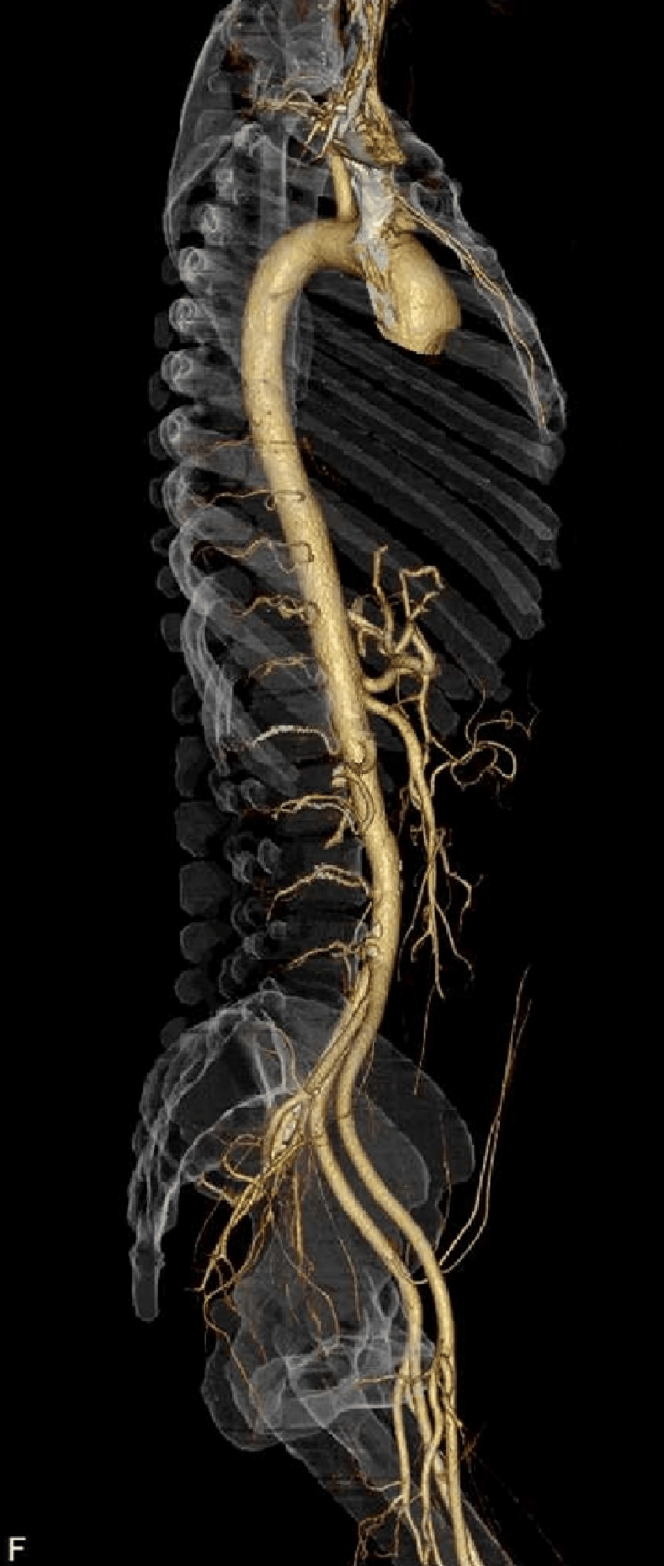
The result of three-dimensional computed tomography (CT) angiography performed on day six. CT angiography shows no abnormalities in the aorta.

Spinal magnetic resonance imaging (MRI) on admission revealed no abnormalities on T2-weighted imaging but showed hyperintensities at the Th11 and Th12 levels on diffusion-weighted imaging (Figure 1). She was diagnosed with spinal cord infarction and treated with intravenous methylprednisolone (1000 mg/day for three days), heparin, and rehabilitation therapy. On day three, contrast-enhanced gadolinium MRI revealed no enhancement, but patchy hyperintensities from Th10 to Th12 on both diffusion-weighted and T2-weighted imaging with ‘owl eye’ appearance were noted (Figure 2). Three-dimensional computed tomography (CT) angiography performed on day six showed no abnormalities in the aorta (Figure 3).

**Figure 4:**
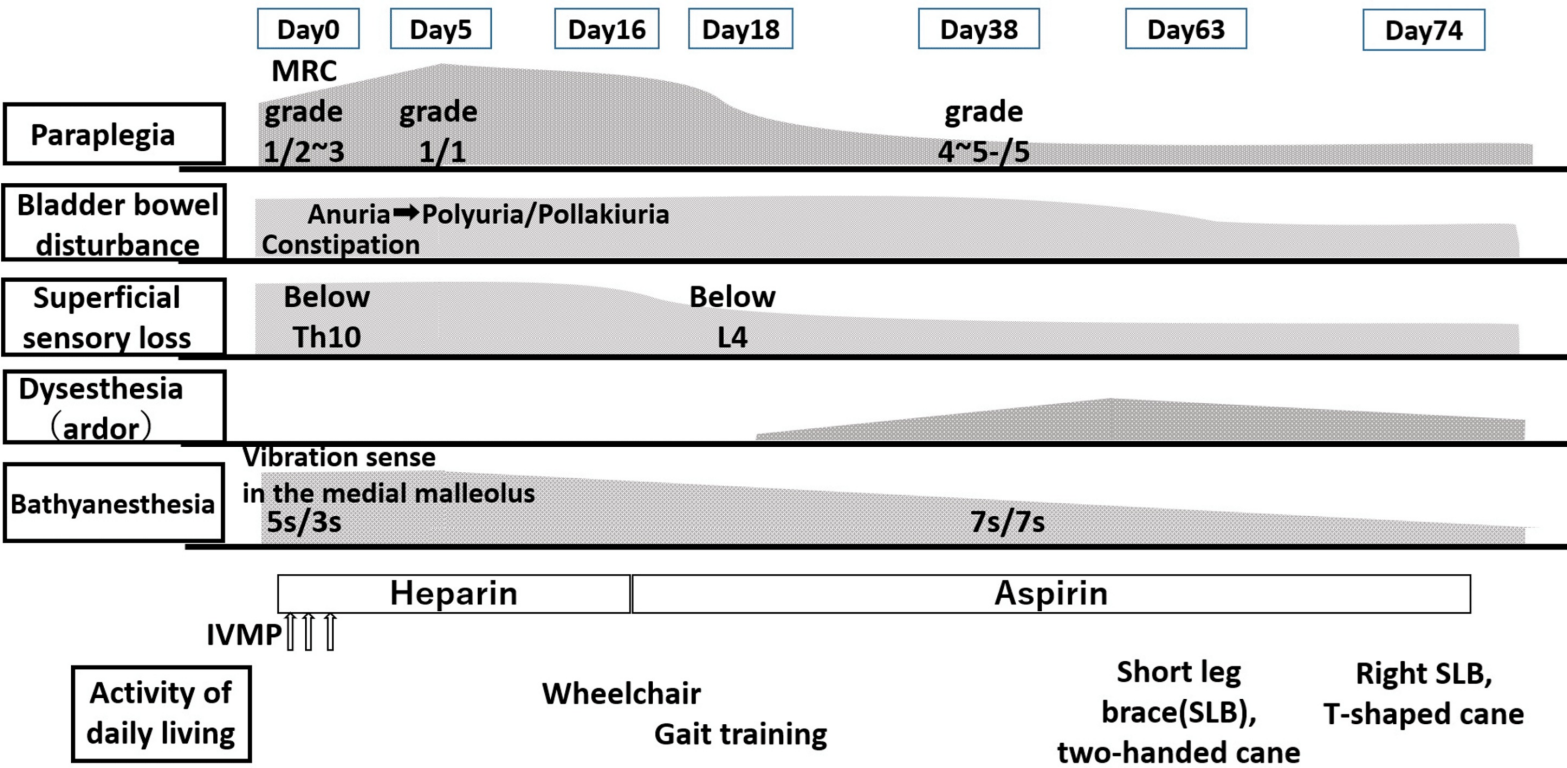
Clinical and therapeutic course diagram. MRC: Medical Research Council; IVMP: intravenous methylprednisolone pulse

The clinical course is illustrated in Figure 4. Several days after admission, she developed dysesthesia, described as burning sensations, in both lower extremities. On day 16, intravenous heparin was switched to oral aspirin. By day 18, superficial sensory loss had improved below the L4 level. By day 38, improvement in her symptoms was observed, with muscle strength increasing to grade four in the right lower extremity and grade five in the left, along with partial recovery of deep sensation. However, dysesthesia persisted. Between days 38 and 50, her bladder and rectal dysfunctions gradually improved, and urinary retention shifted to polyuria. Additionally, medications such as mirabegron, sennoside, macrogol, and magnesium oxide successfully brought her bowel and bladder dysfunctions under control. With rehabilitation therapy, she achieved independent walking with a right short leg brace and a T-cane by day 74 and was subsequently discharged on day 80.

## Discussion

We describe a case of spinal cord infarction (SCI) that resulted in paraplegia, bladder and rectal dysfunction, and pansensory deficits below the Th10 level. Due to the sudden onset and rapid progression of symptoms, a vascular cause was considered more likely than an inflammatory or demyelinating disease. As superficial sensory disturbances were predominantly on the left side, whereas paralysis and deep sensory disturbances were predominantly on the right side, our case resembled Brown-Séquard syndrome. MRI revealed patchy involvement from Th10 to Th12, primarily affecting the areas supplied by the anterior spinal artery. In addition, an ‘owl eye’ appearance was noted. The ‘owl eye’ appearance, also referred to as the snake's eyes sign or fried-eggs sign, describes bilaterally symmetrical round or oval high T2-weighted signals in the anterior horn cells of the spinal cord, visible on axial MR images [5,6]. This pattern is associated with spinal cord infarction due to anterior spinal artery involvement [5,6]. In our case, the urinary issue initially presented as urinary retention but later transitioned to polyuria. Post-spinal cord injury-related polyuria is believed to arise from a loss of vascular tone in the lower extremities, leading to edema [7]. The excess fluid from this edema then contributes to polyuria [7]. Following rehabilitation treatment, the patient showed remarkable improvement in symptoms, but paraplegia, bladder-rectum dysfunction, and pansensory deficits remained.

Our case was notable for being accompanied by pansensory impairment, which is relatively rare among cases of spinal cord infarction (SCI). One reason for this rarity is that ASA primarily supplies blood to areas of the spinal cord other than the posterior columns (i.e., the anterior two-thirds of the spinal cord), while the two PSA primarily supply the posterior columns (the posterior one-third) [4]. Therefore, when either blood vessel is occluded, superficial sensory impairment or deep sensory impairment typically occurs. However, some reports have described pansensory disorders related to SCI. Moreover, previous studies have shown that pansensory impairment persisted in all reported cases [4]. Upon reviewing these reports, we identified three possible mechanisms: the transverse spinal infarction pattern; the Brown-Séquard syndrome pattern; and the edema caused by SCI affecting neighboring tissue patterns.

First, among SCI cases, transverse infarction is the most common pattern, accounting for 52.5% [4]. However, not all cases of transverse spinal cord infarction develop pansensory deficits [4]. Transverse spinal infarction is mainly associated with the Adamkiewicz artery, which is likely to cause longitudinally extensive transverse infarction at the thoracolumbar segment [4]. In general, transverse spinal infarction tends to present as bilaterally symmetrical, uniformly wide infarction on imaging [4,8], and is often caused by embolism [3]. Therefore, cases with elongated, patchy infarction images, like ours, appear to be rare.

Second, there are reports of Brown-Séquard syndrome following vascular SCI [9-11]. Ischemia of the sulco-commissural arteries (central sulcal artery) separated from the anterior spinal arteries or the radicular artery (which forms the spinal coronary artery ring, supplying the surface of the spinal cord and the peripheral region of the parenchyma) may result in partial Brown-Séquard syndrome [9-11]. Our case also presented with partial Brown-Séquard syndrome.

Third, Ferreira et al. reported a case of SCI with pansensory loss following ASA occlusion [12]. They focused on perilesional edema after spinal cord infarction and described its clinical presentation. However, in that case, deep sensory impairment persisted as a residual effect, making it difficult to attribute the condition solely to edema.

In light of these three possible mechanisms for the development of pansensory deficit, our case may have been caused by ischemia of the sulco-commissural arteries or the radicular artery, because our cases manifested Brown-Séquard syndrome type. The MRI findings showed patchy areas of abnormal intensity in the long cord, suggesting an embolic mechanism. However, MRI has its limitations, and the responsible blood vessels could not be identified. Further research is needed to elucidate the underlying mechanisms leading to pansensory deficits related to SCI.

## Conclusions

In this report, we classify three mechanisms of pathogenesis responsible for pansensory deficits associated with SCI. The mechanism may be inferred from both clinical symptoms and imaging findings. Our case could have resulted from ischemia in the sulco-commissural arteries or the radicular artery, given the manifestation of Brown-Séquard syndrome type. Additionally, while sensory impairment persisted as a residual effect in our case, treating pansensory deficits due to SCI remains challenging. Further research is necessary to elucidate the underlying mechanisms that contribute to these deficits in SCI.
